# The Parenting Practices of Parents with Psychosis: A Systematic Integrative Review

**DOI:** 10.1007/s10567-025-00518-6

**Published:** 2025-04-23

**Authors:** Hannah Collins, Anja Wittkowski, Lynsey Gregg

**Affiliations:** 1 Division of Psychology and Mental Health, School of Health Sciences, University of Manchester, Manchester, UK; 2 Greater Manchester Mental Health NHS Foundation Trust, Manchester, UK; 3 Manchester Academic Health Science Centre, Manchester, M13 9NQ UK

**Keywords:** Schizophrenia, Mothers, Fathers, Serious mental illness, Narrative synthesis

## Abstract

Parental psychosis has been reliably associated with adverse outcomes for both parents and children. Despite this, support for these families remains limited. Understanding the everyday parenting practices of parents with psychosis, and whether they differ from parents without psychosis is crucial for developing suitable, evidence-based interventions. We therefore aimed to synthesise quantitative and qualitative research to answer two research questions: (1) ‘What are the parenting practices of parents who experience psychosis?’ and (2) ‘Are the parenting practices of parents who experience psychosis the same as the parenting practices of parents without serious mental illness (SMI)?’ Five databases were searched for terms associated with parenting, psychosis and parenting practices, following the Preferred Reporting Items for Systematic Review and Meta-Analysis guidelines. The Mixed Methods Appraisal Tool was used for quality appraisal prior to an integrative narrative synthesis being conducted. Twelve studies (n = 9 quantitative; n = 3 qualitative) containing 1115 parents with psychosis were included. The synthesis revealed that parents with psychosis frequently use positive authoritative parenting strategies, but sometimes this can be difficult to sustain, with parents resorting to permissive and inconsistent parenting practices. They appear to do so more frequently than parents without SMI but because only four studies utilised a control group, more comparative research is needed. The review recommends further support, and use of parenting interventions for parents with psychosis, alongside systemic practice change initiatives within adult mental health services.

## Introduction

Psychosis is a cluster of symptoms involving disturbed thoughts, loss of contact with reality, delusions, and hallucinations (American Psychiatric Association, 2013). Around one in 150 people will be diagnosed with schizophrenia or a related psychotic disorder in their lifetime (Moreno-Küstner et al., 2018) and between 36 and 44% are parents of dependent children (Campbell et al., 2012; Radley et al., 2022a). Parents with psychosis have described being a parent as enhancing their lives, giving them a sense of purpose, pride and motivation (Ackerson, 2003; Evenson et al., 2008). However, parenting can be particularly challenging for parents with psychosis (Campbell et al., 2012; Wan & Green, 2009). Symptoms, medication side-effects, hospitalisation, stigma and guilt can impact a parent’s ability to parent effectively, engage with their child (Montag et al., 2007), tune into their child’s needs (Mehta et al., 2014; Wan et al., 2007) and be available emotionally and physically for their child (Somers, 2007; Strand et al., 2020).

Parents with psychosis are more likely to have a smaller social network and experience housing difficulties, unemployment and be single parents (Boydell et al., 2013; Craig & Bromet, 2004; Kessler et al., 1995; Topor et al., 2016). As a result, their children are more likely to experience social adversity, have poorer emotional and behavioural outcomes (Davidsen et al., 2022; Dean et al., 2010; Somers, 2007), and many develop their own mental health difficulties by early adulthood (Rasic et al., 2014; Riches et al., 2019). Despite this, little is known about how parents with psychosis parent and what support may be necessary to support families in which a parent has psychosis. Consequently, parental needs are often dismissed and overlooked, leaving these parents feeling ashamed, guilty and unsupported by services (David et al., 2011; Goodyear et al., 2022; Harries et al., 2023a).

Over the last decade, research exploring the experiences of parents with psychosis has increased, accompanied by policy guidance for those experiencing serious mental illness (SMI; Bee et al., 2014; Diggins, 2011; Foster et al., 2019; Reedtz et al., 2021). However, the exploration of parenting practices has been scarce, with studies primarily focusing on parental experiences (Harries et al., 2023a), parent–child interaction with infants (Mullick et al., 2001; Wan et al., 2007), support for children or other family members (Fekadu et al., 2019; Radley et al., 2023) or gained only a retrospective view from the adult or adolescent child’s perspective on their parents’ parenting practices (Cierpiałkowska et al., 2021; Herbert et al., 2013; Källquist & Salzmann‐Erikson, 2019). The UK’s Social Care Institute for Excellence (SCIE) guidelines have recognised the increased probability of parents with mental health difficulties being marginalised from health and social care services, emphasising the need for broader initiatives to help engage these vulnerable families (Department of Health, Social Services and Public Safety, ≥; Diggins, 2011). Family-focused practice aims to meet the needs of parents with SMI and their children (Foster et al., 2016; Reupert et al., 2015), and has been found to mitigate the risk of adverse outcomes (Foster et al., 2016; Grant et al., 2019; Maybery et al., 2015); yet Furlong et al. (2021) stressed the inadequate implementation of these approaches, with a recent systematic review highlighting the challenges associated with their integration into service provision (Tuck et al., 2023). Considering the influence of parental practices on children’s outcomes (Kahng et al., 2008), gaining a deeper understanding of the parenting practices of parents with psychosis is crucial for improving support for this vulnerable group.

Much of the research involving parents with SMI has grouped parents with different diagnoses together (Fekadu et al., 2019; Harries et al., 2023b). In a mixed review of 67 studies of mothers with SMI conducted by Oyserman et al. (2000) most focused on low mood or depression, with only ten studies recruiting any parents with a diagnosis of psychosis. Given that psychosis symptoms can often be intermittent and unpredictable, and parents with psychosis tend to experience greater social adversity, they may have different needs and challenges to parents with other diagnoses (Evenson et al., 2008). Therefore, it is important to explore what research has been carried out specifically investigating the parenting practices of parents with psychosis.

A review by Engur (2017) purported to focus on the impact of psychosis on parenting but descriptions of the seven included studies were insufficient and results were not synthesised, meaning that any conclusions drawn were not robust. Thus, there is a clear need for a more robust and up to date review focusing solely on the parenting practices of parents with psychosis. Therefore, this mixed method narrative synthesis aimed to synthesise the literature exploring the parenting practices of parents with psychosis, to identify gaps in the literature and guide future research and clinical practice. We specifically addressed the following questions: (1) What are the parenting practices of parents who experience psychosis? (2) Are the parenting practices of parents who experience psychosis the same as the parenting practices of parents without SMI?

## Methods

This mixed methods systematic review and narrative synthesis (Popay et al., 2006) was informed by the preferred Reporting Items for Systematic Reviews and Meta-Analysis guidelines (PRISMA; Page et al., 2021). The review protocol was registered with PROSPERO on 14/03/2024 (CRD42024524465).

### Search Strategy

The search was conducted electronically using five databases relevant for this topic area: PsycINFO, CINAHL Plus, Medline, EMBASE and Web of Science on 27/03/2024 and was updated on 04/11/2024. Search terms were informed by the titles and abstracts of recent key papers and reviews in the same field (e.g., Strand et al., 2020; Radley et al., 2020, 2022b) to ensure comprehensive parenting-related terms were included. Pilot searches were undertaken to help generate the final search terms (see Table 1). Medical Subject Heading (MeSH) terms were used to identify synonyms and Boolean operators (“AND”, “OR”) were used to combine terms and concepts. The reference lists of included studies were also searched (Horsley et al., 2011).

**Table 1:** Search strategy and terms

Database (and platform)	PsycInfo (OVID); Medline (OVID), EMBACE (OVID), CINAHL Plus (EBSCOhost); and Web of Science (Clarivate)
1	(Parent* or Mother* or Father* or Caregiv* or Guardian* or Carer* or Kinship or Stepparent* or Foster parent*)
2	(Psychos* or schizo* or psychotic or hallucin* or paranoi* or voice hear* or psychiatric* or unusual belief* or thought disorder*)
3	(Parent* practices or Parent* style* or Parent* beh* or Parenting)
4	1 AND 2 AND 3

References from each database were exported to Rayyan (Ouzzani et al., 2016) and duplicates were automatically removed. Titles, keywords, and abstracts were assessed for eligibility against the inclusion and exclusion criteria and retained studies were subjected to a full-text review by the first author. A second independent reviewer (NA) assessed a sample of approximately 5% of the total number of studies for inclusion. Agreement between reviewers was substantial (98.15%, κ = 0.705) and any discrepancies were resolved through discussion. All authors discussed the final selection of papers and agreed their inclusion.

### Inclusion and Exclusion Criteria

Studies were included if they (1) were available in English, (2) used quantitative, qualitative or mixed methods, (3) were published in a peer-reviewed journal, (4) involved parents (mothers, fathers, stepparents, guardians, adoptive parents, foster parents, or kinship parents) with a stated diagnosis of schizophrenia spectrum or other non-affective psychotic disorder and (5) focused on parenting practices. Papers were excluded if the study solely focused on parent–child interaction, exclusively focused on staff, or adult or child offspring perceptions of parenting practices or utilised a sample in which the results for parents with psychosis could not be distinguished from parents with other diagnoses.

### Methodological Quality Assessment

Studies were assessed for methodological quality and risk of bias using the Mixed Methods Appraisal Tool (MMAT; Pluye et al., 2009; Hong, 2018), because it allows for the comparison of different types of study designs (Crow & Sheppard, 2011) and has been demonstrated to have good reliability and validity (Pace et al., 2012; Souto et al., 2015).The MMAT contains 19 methodological criteria and is split into five categories (1) qualitative research, (2) randomised controlled trials, (3) non-randomised studies, (4) quantitative descriptive studies and (5) mixed methods studies, with five items specific to the study’s methodology and two screening items appropriate for all studies. These criteria are scored according to the MMAT evaluation criteria and rated as ‘yes’, ‘no’ or ‘can’t tell’. Each paper was provided an overall quality appraisal as high, moderate, or low based on the total number of items rated ‘yes’ for the core five questions [high quality (4 or 5 items rated ‘yes’) = 80–100%; moderate quality (2 or 3 items rated ‘yes’) = 60–40%; low quality (1 item rated ‘yes’) = < 40%]. Each study was independently rated by the first author and 50% by an independent reviewer (NA). Substantial agreement was achieved between the two reviewers (90.16%, κ = 0.72). Any disparities in ratings were discussed, mutually agreeing on a final rating.

### Data Extraction and Analysis

As part of the synthesis, relevant data were extracted from studies and tabulated according to study methodology (quantitative or qualitative) and ordered by date, with studies reporting data from the same sample placed next to each other in the table. A narrative synthesis of the findings was conducted following Popay et al.’s (2006) adjusted systematic steps: (1) developing a preliminary synthesis, (2) exploring the relationships within and between studies and (3) appraising the robustness of the synthesis. Data were synthesised and categorised by the two research questions. Quantitative and qualitative studies were initially analysed separately, but data synthesis occurred simultaneously, in line with the Mixed Methods Systematic Review integrative data-based convergent approach (Stern et al., 2020).

The synthesis identified similarities and differences between the methodologies used to explore or investigate parenting practices, followed by an exploration of parenting practices, carried out by comparing studies that used similar parenting measures and organised/grouped by parenting style. Parenting practices were grouped in accordance with Baumrind’s (1966) three parenting styles i.e. authoritative (positive) parenting, permissive (inconsistent) parenting and authoritarian parenting, because these aligned well with the data collection methods used and the data presented in the included studies. Finally, studies utilising a control group were synthesised and a comparison between the parenting practices of those experiencing psychosis and those without SMI were made.

## Results

**Fig. 1:**
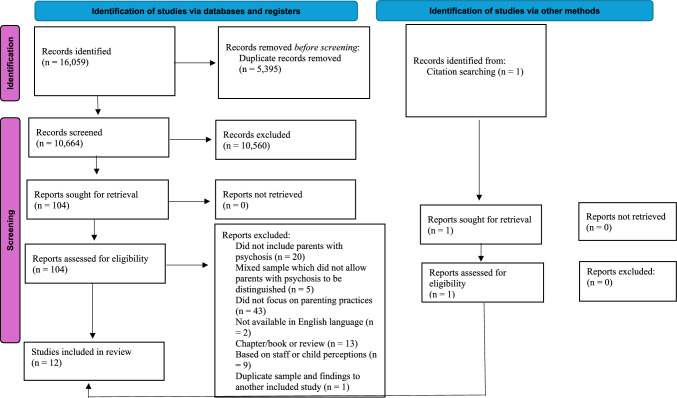
PRISMA (2020) flow diagram outlining screening process

A total of 13,725 records were identified by five databases, with 5095 duplicates. After screening title and abstracts, 90 full-text articles were reviewed. One additional study was included through citation searching and a total of 12 studies were included in the review. An outline of the systematic search process is illustrated in Fig. 1 and an overview of the studies in presented below.

### Study Characteristics

Twelve studies (n = 9 quantitative and n = 3 qualitative) were identified and synthesised (Fig. 1). Characteristics of each included study are presented in Table 2. Two studies (Graham & King, 2005; Wolfenden et al., 2022) used a mixed-methodological approach. As only the qualitative or quantitative aspect of the studies was relevant to this review, for the purpose of this review they were categorised as either a qualitative (Graham & King, 2005) or quantitative (Wolfenden et al., 2022) study. Furthermore, through screening, it was recognised that Sabih et al. (2023) presented the same data as an earlier study (Sabih et al., 2020). As the additional information in the earlier study was not relevant to the current research questions, it was excluded from the review.

**Table 2:** Characteristics of the included 12 studies

	Study: authors, year, location and aim	Design	Sample description			Recruitment method	Relevant data collection
			Parental mental health difficulty and verification of diagnosis	Child age	Other socio-demographical information		
Quantitative (n = 9)							
1	Sabih et al. (2023), Pakistan To examine parenting practices and differences in behavioural problems of adolescents	Cross-sectional	n = 348 families n = 173 parents with SMI (n = 66 Schizophrenia and n = 107 major depression disorder; MDD); verified by DSM-5 diagnosis Control group: n = 175 without SMI	12–18 years	Mothers (n = 26); fathers (n = 40) with schizophrenia Age (years): *M* = 42.66 Ethnicity: Not stated Household composition: Dual family household (n = 348) Family status: Not stated	Psychiatric inpatient units and outpatient clinics	Alabama Parenting Questionnaire Parent and Child Form (APQ, Shelton et al., 1996; Urdu version, Mushtaq, 2015)
2	Rabha et al. (2021), India To evaluate and compare parenting skills	Cross-sectional	n = 51 parents with schizophrenia; verified by DSM-IV diagnosis and their children Control group: n = 51 parents without SMI	15–20 years	Mothers (n = 22), fathers (n = 29) with schizophrenia Age (years): *M* = 45.23 Ethnicity: Not stated, completed in India Household composition: Dual (n = 27), extended/joint (n = 24) Family status: Not stated	Not stated	APQ (Shelton et al., 1996)—both parent and child form
3	Gregg et al. (2021), UK To better understand the emotional climate of families	Cross-sectional	n = 20 parents with schizophrenia; verified by ICD-10 checklists and case note review Control group: n = 20 parents without SMI (n = 19 mothers; n = 1 father)	3–11 years	Mothers (n = 19) fathers, n = 1) Age (years): *M* = 33.9 Ethnicity: White (n = 15); Black (n = 2); Chinese (n = 1); South Asian (n = 1); Mixed (n = 1) Household: composition: Dual (n = 5), single (n = 15) Family status: Not stated	Community Mental Health Teams (CMHT’s), psychosis outpatient clinics and local authority services	– The Parenting Scale (PS; Arnold et al., 1993) – The Parenting and Family Adjustment Scales (PAFAS; Sanders et al., 2014)
4	Shenoy et al. (2019), India To assess parenting and examine the clinical correlates of parenting	Cross-sectional, observational study	n = 50 mothers with schizophrenia; verified by DSM-IV diagnosis Control group: n = 50 mothers without a psychiatric disorder	3–11 years	Mothers (n = 50) Age (years): *M* = 33.6 Ethnicity: Not stated, completed in India Household composition: Not stated Family status: Unclear, stated 18% of psychosis sample separated from partner	Psychiatric outpatient clinics	PS Revised (Rhoades & O’Leary, 2007)
5	Campbell et al. (2018), Australia To identify predictors of positive parenting outcomes	Two-phase design	n = 234 parents with psychosis; verified by ICD-10 diagnosis	Under 18 years	Mothers (n = 174), fathers (n = 60) Age (years): *M* = 37 Ethnicity: Not stated Household composition: Not stated Family status: Single (n = 113); married (n = 121)	Data from Australian national survey of psychosis (Morgan et al., 2012)	Study specific survey and Interview exploring quality of care in the last 12 months
6	Campbell et al. (2012), Australia To report on parental experiences	Two-phase design	n = 448 parents with psychosis; verified by ICD-10 diagnosis	Under 18 years	Mothers (n = 253); fathers (n = 195) Age (years): Mother (*M* = 36.94); fathers (*M* = 38.15) Ethnicity: not stated Household composition: Not stated Family status: Single (n = 287); married (161)	Data from Australian national survey of psychosis (Morgan et al., 2012); same as above	Study specific survey and interview-explored quality of care over last 12 months
7	Mowbray et al. (2002), USA To examine parenting among mothers who vary in demographic and symptoms	Cross-sectional	n = 379 mothers with SMI; n = 35 schizophrenia, n = 34 schizoaffective, n = 40 MD with psychotic features, n = 53 BD with psychotic features, n = 134 MD, n = 41 BD; verified by DSM-IV diagnosis	4–16 years	Mothers (n = 379) Age (years): 36.2 Ethnicity: African American (n = 228); White (n = 110); Hispanic (n = 30); Other (n = 11) Household composition: Not stated Family status: Not stated	12 CMHT’s and 3 psychiatric inpatient units	Nurturance, parental nurturance subscale of the Child Rearing Practices Report (Rickel & Biasatti, 1982)
8	Zemencuk et al. (1995), USA To better understand the mothers functioning and parenting style	Cross-sectional	n = 48 mothers with a diagnosis of schizophrenia (65% schizophrenia, 13% schizoaffective, 21% affective); verification of diagnosis not stated but recruited from psychiatric inpatient units	Under 13 years	Mothers (n = 48) Age (years): *M* = 31.4 Ethnicity: Black (n = 31), White (n = 17) Household composition: Not stated Family status: Single (n = 38), married (n = 10)	4 Psychiatric inpatient units	STC (Stollak et al., 1973)—responses rated to 5 vignettes
9	Wolfenden et al. (2022), UK An evaluation of Triple P parenting intervention	Within subject ABA single case design	n = 10; n = 6 schizophrenia, n = 4, paranoid schizophrenia; verified by ICD-10 diagnosis	3–10 years	Mothers (n = 10) Age (years): *M* = 32.9 Ethnicity: White British (n = 8), Black African (n = 1), Chinese (n = 1) Household composition: Single (n = 9), dual (n = 1) Family status: Single (n = 9), cohabiting (n = 1)	CMHT’s, psychosis outpatient services, local authority services	PS (Arnold, 1993)

	Study: authors, year, location and aim	Design	Sample description			Recruitment method	Relevant data collection and analysis
			Parental mental health difficulty and verification of diagnosis	Child age	Other socio-demographical information		
Quantitative (n = 3)							
10	Boström and Strand (2021), Sweden To explore parent and child mental health and the parent–child relationship	Multi perspectival design	n = 6 parents with psychosis and their 7 children; verification of diagnosis not stated, but recruited from psychosis outpatient clinics	8–15 years	Mothers (n = 4), fathers (n = 2) Age (years): *M* = 44.3 Ethnicity: Swedish (n = 5), other (n = 1) Household composition: Single (n = 4), dual (n = 2) Family status: Single (n = 4), co-habiting (n = 2)	4 Psychosis outpatient clinics Parents participated in Beardslee family intervention (Beardslee et al., 2003)	Semi-structured interviews; analysed using IPA (Smith et al., 2009)
11	Strand et al. (2020), Sweden To explore how parents experience the effect(s) of their illness on parenting	Exploratory study	n = 15 parents with psychosis; n = 8 schizoaffective disorder, n = 2 schizophrenia, n = 3 psychotic disorder and n = 2 MDD with psychotic episodes; verification of diagnosis not stated, but recruited from psychosis outpatient clinics	2–16 years	Mothers (n = 10), fathers (n = 5) Age (years): *M* = 42 Ethnicity: Not stated Household composition: Single (n = 7), dual (n = 8) Family status: Single (n = 7), co-habiting or married (n = 8)	Psychosis outpatient clinics	Semi-structured interviews; parenting was analysed using Grusec and Davidov’s (2010) model of parenting
12	Graham and King (2005), Australia To explore potential parental difficulties during their child’s bedtime routine	Cross-sectional, descriptive	n = 5 mothers with schizophrenia; verification not stated, but recruited via case managers at psychiatric clinic	3–12 years	Mothers (n = 5) Age (years): *M* = 33.4 Ethnicity: Not stated Household composition: Not stated Family status: Single (n = 3), co-habiting or married (n = 2)	2 CMHT’s	Semi-structured interviews; analysed using thematic analysis (Aronson, 1994)

The 12 included studies were conducted between 1995 and 2023 across six different countries. One thousand one hundred and fifteen parents^1^ diagnosed with psychosis appeared to be included in the studies, with five studies reporting on only mothers’ parenting practices. All studies detailed age and sex, and most studies commented on marital status, however, less than half (n = 5) reported ethnicity and half did not report on household composition. Of the six studies that did report household composition, 79% of parents reported living with another adult, and the remainder resided only with their children.

Sample sizes varied across study designs, with quantitative studies ranging from just 10 parents with psychosis to 448. Qualitative studies ranged from 5 to 15 parent participants. The age range of the child varied throughout the studies: four studies included parents of children who were over the age of 16, stating inclusion of a wide child age range (from ages 1 through to 21). Five studies focused upon younger children and included parents of children aged 3–13. Additionally, only four studies utilised a control group.

Diagnoses were verified by the Diagnostic and Statistical Manual of mental disorders, fourth edition (DSM-IV; American Psychiatric Association, APA, 1994; n = 2), the DSM-IV-text revised (DSM-IV-TR; APA, 2000; n = 3) or the International Classification of Diseases, 10th edition (ICD-10; World Health Organisation, WHO, 1993; n = 4). Four studies, including all three of the qualitative studies, did not state clearly how diagnosis was verified, but all four samples were recruited from psychiatric services or mental health teams/organisations.

Studies used various ways to explore and investigate the parenting practices of parents with psychosis. Nine of the quantitative studies relied on five different validated measures, with the remaining two studies utilising study-specific surveys and interviews. The most frequently used measure was the *Parenting Scale* (PS; Arnold et al., 1993), which was used in three of the studies, followed by the *Alabama Parenting Questionnaire* (APQ; Shelton et al., 1996) used by two studies. Semi-structured interviews were used in all three of the qualitative studies, with two using thematic analysis to analyse the data and one using interpretative phenomenological analysis.

#### Research Question 1: What Are the Parenting Practices of Parents Who Experience Psychosis?

As presented in Table 3, parenting practices were grouped in accordance with Baumrind’s (1966) three parenting styles i.e. authoritative (positive) parenting, permissive (inconsistent) parenting and authoritarian parenting because the most frequently used data collection measure Arnold et al.’s Parenting Scale was based on Baumrind’s (1966) model of parenting styles.

**Table 3:** Summary of key findings in relation to research questions

	Study: authors, year	Findings				Quality appraisal
		Positive Authoritative Parenting	Permissive/Inconsistent Parenting	Authoritarian Parenting	Factors impacting on parental role/additional findings	
Quantitative (n = 9)						
	Measure: Alabama Parenting Questionnaire (Shelton et al., 1996; n = 2); Higher scores = increased frequency of parenting style					
1	Sabih et al. (2023)	Parents with psychosis reported significantly less positive involvement/parenting (*M* = − 1.48; SD = 1.46) than parents without SMI (*M* = 1.13; SD = 1.49)	Parents with psychosis scored significantly higher on poor monitoring/supervision (*M* = 0.42; SD = 1.72) compared to parents without SMI (*M* = − 0.33; SD = 1.67)	Parents with psychosis scored significantly higher on negative/inconsistent discipline (*M* = 0.8; SD = 1.97) compared to parents without SMI (*M* = − 0.47; SD = 1.67)		High
2	Rabha et al. (2021)	Parents with psychosis reported significantly less positive involvement (*M* = 1.93; SD = 0.73) and positive parenting (*M* = 1.91; SD = 0.76) than parents without SMI (Involvement; *M* = 2.89; SD = 0.73); (Parenting; *M* = 3.30; SD = 0.74) Parents with psychosis scored highest on the subscale positive involvement followed by positive parenting when compared to the other subscales	Parents with psychosis scored significantly higher on poor monitoring/supervision (*M* = 1.35; SD = 0.44) and inconsistent discipline (*M* = 1.82; SD = 0.51) compared to parents without SMI (Monitoring/supervision; *M* = 0.70; SD = 0.44); (Inconsistency; *M* = 1.37; SD = 0.66)	No significant differences found between groups on corporal punishment (*M* = 1.3; SD = 0.81); Control (*M* = 1.13; SD = 1.23)		Moderate

	Measure: Parenting Scale (PS; Arnold et al., 1993; n = 3); Higher scores = increased frequency of parenting style and The Parenting and Family Adjustment Scale (PAFAS; Sanders et al., 2014; n = 1); Higher scores indicate more inconsistency, coercion, less positive encouragement and worse parent–child relationship					
3	Wolfenden et al. (2022)	PS: Not measured	PS: Baseline scores - Parental Laxness: *M* = 4.625, SD 1.796	PS: Baseline scores- Parental over-reactivity: *M* = 3.875, SD = 1.081 Parental verbosity: *M* = 4.20, SD = 0.60	PS: At baseline 8 out of 10 parents PS scores fell within the clinical range (*M* = 4.06, SD = 0.77)	High
4	Gregg et al. (2021)	PAFAS: No significant difference was found between the groups on positive encouragement (Psychosis; *M* = 5.55; SD = 3.35); (Control; *M* = 3.8; SD = 3.27)	PS: Parents with psychosis scored significantly higher on parental laxness (*M* = 4.50; SD = 1.63), compared to parents without SMI (*M* = 2.35; SD = 0.77) PAFAS: Parents with psychosis scored significantly higher on parental inconsistency (*M* = 8.7; SD = 2.7) compared to parents without SMI (*M* = 5.1; SD = 2.44)	PS: Parents with psychosis scored significantly higher on parental over-reactivity (*M* = 3.63; SD = 1.41) compared to parents without SMI (*M* = 2.54; SD = 0.70), as well as on parental verbosity (Psychosis; *M* = 4.56; SD = 1.02); (Control; *M* = 3.91; SD = 0.54) PAFAS: Parents with psychosis scored higher on poorer parent–child relationship (*M* = 8.35; SD = 2.51) compared to parents without SMI (*M* = 4.75; SD = 2.81) No significant difference was found between the groups on parental coercion (Psychosis; *M* = 7.65; SD = 3.82); (Control; *M* = 5.9; SD = 3.7)	Lower parental self-efficacy linked to over-reactivity	High
5	Shenoy et al. (2019)	Not measured	PS: Parents with psychosis scored significantly higher on parental laxness (*M* = 3.92; SD = 1.50), compared to parents without SMI (*M* = 2.64; SD = 1.05)	PS: No significant differences found between the groups on parental hostility (Psychosis; *M* = 3.10; SD = 1.59); (Control; *M* = 2.60; SD = 1.35) or over-reactivity (Psychosis; *M* = 3.18; SD = 1.05); (Control; *M* = 3.20; SD = 1.08)	Negative symptoms, lack of judgement/insight and difficulties recognising emotions correlated with laxness	Moderate

	Measure: Study specific survey and interview (n = 2); Rated as no dysfunction, obvious dysfunction or severe dysfunction					
6	Campbell et al. (2018)	75% of parents were rated as having no dysfunction in their quality of care over the last 12 months	Not measured	Not measured	– Majority of parenting is affected by illness severity and daily functioning – Parents valued their role as parents and the majority are parenting well	High
7	Campbell et al. (2012)	76.6% of parents were rated as having no dysfunction in their parenting over the past 12 months	Not measured	Not measured	21.3% of mothers and 28.3% of fathers were rated as having obvious or severe dysfunction in their ability to care for their child	High

	Measure: The Sensitivity to Children Scale (Stollak et al., 1973; n = 1); Higher scores = higher number of responses					
8	Zemencuk et al. (1995)	An authoritative response was the predominant in 3/5 vignettes (1, 2 and 3) Vignettes 1–5 solutions: Authoritative (38.3%, 40.4%, 31.9%, 21.3%, 27.7%)	A neglecting response was most prevalent alongside authoritarian in vignette 4 Vignettes 1–5 solutions: Neglecting (19.1%, 19.1%, 25.5%, 25.5%, 12.8%) Indulgent (23.4%, 4.3%, 23.4%, 23.4%, 4.3%)	An authoritarian response was most prevalent in vignette 5 and authoritarian and neglectful most prevalent in vignette 4 Vignettes 1–5 solutions: Authoritarian (10.6%, 29.8%, 14.9%, 25.5%, 51.1%) Psychotic/intrusive responses were rare (from 4.3 to 8.5%)		High

	Measure: Child Rearing Practices Report (Rickel & Biasatti, 1982; n = 1); Higher scores = more nurturance					
9	Mowbray et al. (2002)	Among non-African Americans, mothers with schizoaffective disorder had significantly lower nurturance scores than those with MD, MD—psychotic features, or BD (Schizophrenia: *M* = 3.18; Schizoaffective: *M* = 3.46; MD with psychotic: *M* = 3.77; BP with Psychotic: *M* = 3.66; MD: *M* = 3.68; BP: *M* = 3.77) Among African Americans, mothers with schizoaffective disorder were significantly less nurturing than those with BP (Schizophrenia: *M* = 3.73; Schizoaffective: *M* = 3.57; MD with psychotic: *M* = 3.61; BP with Psychotic: *M* = 3.72; MD: *M* = 3.61; BP: *M* = 3.80)	Not measured	Not measured	For most of the parenting variables, the results suggest that African American women with a schizoaffective diagnosis and non-African American women with schizophrenia or schizoaffective diagnoses had more parenting problems than women with other diagnoses	High

Qualitative (n = 3)						
	Study: authors, year	Main themes	Summary of main relevant findings			Quality appraisal
10	Boström and Strand (2021)	Five main themes: (1) An unclear image (2) An incoherent story (3) Illness as part of ordinary life (4) A non-hierarchical parent–child relationship (5) Attunement of the parent–child relationship and child well-being	**Positive authoritarian:** Parent–child relationships seemed to be mutually caring and warm **Permissive/Inconsistent:** – Both parents and children described their parenting as permissive, exercising little rules or controlling parenting behaviour – Children described reverse roles and taking responsibility as the parent could not always be relied upon			High
11	Strand et al. (2020)	Domains of parenting: (1) Protection (2) Reciprocity (3) Control (4) Guided learning (5) Group participation	**Permissive/inconsistent parenting:** Parents reported difficulties with control and described using withdrawal and avoidance to help protect their child from their mental illness **Factors impacting on parenting/additional findings:** – Found that all domains of parenting appeared to be affected by psychosis – Depression, fatigue, and difficulty focusing because of hearing voices had negative impacts on parents abilities to provide protection, reciprocity, and control			High
12	Graham and King (2005)	Three themes: (1) Bedtime strategies and effectiveness (2) Maternal responsiveness (3) Understanding of the child’s experience of bedtime	**Factors impacting on parenting/additional findings:** – Mothers demonstrated a poor understanding of their child's bedtime anxiety – Parents described difficulty being effective with bedtime strategies and attributed it to medication-induced fatigue (reduced responsiveness and difficulty waking in the night)			High

##### Positive Authoritative Parenting

Of the 12 studies, 7 measured or commented on positive aspects of parenting, including positive involvement/parenting, positive encouragement and offering nurturance and warmth to the child.

Utilising the Alabama Parenting Questionnaire, Rabha et al. (2021) found that parents with psychosis were more likely to prioritise positive parenting strategies over permissive or authoritarian methods. Similarly, when parents were asked to rate their solution/response to five vignettes in Zemencuk et al.’s (1995) study, they too rated the most common response as an authoritative (i.e. positive) approach.

Two studies with large sample sizes concluded that parents with psychosis were parenting well, finding that over 75% of parents had no dysfunction in their parenting (Campbell et al., 2012, 2018), although it is not clear what specific parenting practices parents were utilising. Gregg et al. (2021), whose sample consisted of mainly single parents, noted that parents with psychosis often reported providing positive encouragement to their children and found no significant difference to parents without SMI. However, Mowbray et al. (2002) reported lower levels of nurturance among parents with psychosis when compared to other SMI diagnoses.

Only one qualitative study (Boström & Strand, 2021) highlighted positive parenting strategies, and it is unclear whether this was due to the research aims and topics explored/questions asked or whether parents preferred to focus on the difficulties of parenting in the other studies. Whilst exploring the parent–child relationship, Boström and Strand (2021) found that parents with psychosis described their relationship as being mutually warm and caring.

##### Permissive/Inconsistent Parenting

Permissive and inconsistent parenting was measured or explored in eight studies and was most frequently reported on when compared to other parenting practices. In three studies using the Parenting Scale in the UK and India, parents with psychosis consistently scored above the clinical cut-off for parental laxness (Gregg et al., 2021; Shenoy et al., 2019; Wolfenden et al., 2022). Similarly, when parenting practices were explored through interviews with parents with psychosis in Sweden, parents described finding boundary setting difficult (Bostrõm & Strand, 2021; Strand et al., 2020). A parent in Strand et al.’s (2020) study described how their children would take advantage of them when they were feeling fatigued “He knows when I don’t have the energy to discuss [something]. He thinks, ‘If I ask Mum if I can take a sweet, she will say yes’” (p. 625), whilst a mother in Bostrom and Strand’s (2020) paper described herself as “a mother with no rules” (p. 72).

Sabih et al. (2023) and Rabha et al. (2021) both utilised the Alabama Parenting Questionnaire with parents in Pakistan and India to measure permissive/inconsistent parenting, for which a clinical cut-off is not provided. Rabha et al. (2021) found that poor monitoring/supervision and inconsistent discipline were less frequently reported than positive parenting strategies, whereas Sabih et al. (2023) found that parents were more likely to report using authoritarian methods, followed by poor monitoring/supervision. However, as Sabih et al. (2023) grouped the inconsistent discipline and corporal punishment subscales together, it is unclear whether inconsistent discipline or corporal punishment were endorsed more frequently in parents with psychosis in this sample. Similarly, to Rabha et al. (2021), Zemencuk et al. (1995) found that when parents responded to parenting vignettes, most parents used an authoritative response over an indulgent solution in four of the five vignettes.

Parents described different reasons for employing permissive parenting strategies, including using withdrawal and avoidance to help protect their children from their mental health difficulties or giving in due their own anxiety: “When I was psychotic, I stayed away for long periods. I didn’t want her to see me in such bad shape.” (p. 623, Stand et al., 2020). Additionally, Boström and Strand (2021) found that children of parents with psychosis took on the parental role (a process of role reversal sometimes known as ‘parentification’) because they felt their parents could not always be relied upon.

##### Authoritarian Parenting

Authoritarian parenting was measured in six studies which included subscales such as corporal punishment, hostility, over-reactivity and coercive parenting. The three qualitative studies did not explore or report on authoritarian strategies.

Among studies using the Parenting Scale, one study (Wolfenden et al., 2022) reported scores falling above the clinical cut-off on the subscale for over-reactivity; including threats and physical punishment, whereas Gregg et al. (2021) and Shenoy et al. (2019) reported mean scores falling just below the cut off. As Shenoy et al. (2019) utilised a revised version of the PS, an overall score for hostility was reported, which fell above the clinical cut-off. Gregg et al. (2021) and Wolfenden et al. (2022) reported on parental verbosity (the extent to which parents give lengthy verbal reprimands) and found scores also fell above the clinical cut-off.

Rabha et al. (2021) noted that corporal punishment was least likely to be reported by parents with psychosis and was scored the lowest when compared to positive and permissive parenting strategies. Unfortunately, as discussed above, corporal punishment scores could not be distinguished within Sabih et al.’s (2023) study. Within Zemencuk et al.’s (1995) study, they highlighted that an authoritarian solution was the main response in two of the five vignettes.

##### Factors Impacting on the Parenting Role and Additional Findings

Although included studies ranged in child age and family composition, and were conducted in different countries and cultures, there did not appear to be any differences between the studies in relation to these factors. Fatigue, medication side-effects and mental health symptoms were reported to have a negative impact on parents with psychosis and their ability to carry out their parenting role in two of the three qualitative studies (Graham & King, 2005; Strand et al., 2020). Additionally, two quantitative studies (Campbell et al., 2018; Shenoy et al., 2019) stressed the impact of psychosis symptoms and mental health functioning on different aspects of parenting, with Shenoy et al. (2019) finding a significant association between negative symptoms and parental laxness. Furthermore, lower parenting self-efficacy has also been found to be associated with parental practices (Gregg et al., 2021).

#### Research Question 2: Are the Parenting Practices of Parents Who Experience Psychosis the Same as the Parenting Practices of Parents Without SMI?

Out of the 12 studies, only four included a control group of parents without an SMI (Sabih et al., 2023; Rabha et al., 2020; Gregg et al., 2021; Shenoy et al., 2019). These studies utilised three different parenting measures, reporting on Mean and SD scores for each group.

Sabih et al. (2023) and Rabha et al. (2021) both utilised the Alabama Parenting Questionnaire (Sheldon et al., 1996) and yielded similar results, with parents with psychosis reporting significantly less positive involvement and parenting, poorer monitoring and supervision and more inconsistent discipline when compared to parents without SMI. However, Rabha et al. (2021) did not find a significant difference between the groups on corporal punishment.

The Parenting Scale (Arnold et al., 1993) was used by Gregg et al. (2021) and a revised version which assessed parental hostility instead of verbosity was used by Shenoy et al. (2019). Both studies found that parents with psychosis were more likely to report permissive/lax parenting strategies than those without an SMI. However, whereas Gregg et al. (2021) found an increase in reporting of over-reactivity by parents with psychosis, Shenoy et al. (2019) reported no difference between the groups. Additionally, Shenoy et al. (2019) found no difference between the groups on parental hostility, and Gregg et al. (2021) found parents with psychosis were significantly more likely to report parental verbosity. Gregg et al. (2021) also utilised the Parenting and Family Adjustment Scales in addition to the Parenting Scale, finding more inconsistent parenting and a poorer parent–child relationship among parents with psychosis, but no significant differences between the groups with regard to coercion or positive encouragement.

While there were some slight differences between the studies, all four studies (Gregg et al., 2021; Rabha et al., 2021; Sabih et al., 2023; Shenoy et al., 2019) noted that parents with psychosis were more likely to report using a permissive and inconsistent parenting style when compared to parents without an SMI; however, the use of more authoritarian and harsh parenting styles appeared to be less consistent and unclear. Although the Parenting Scale does not measure positive parenting strategies, two out of the three studies that used the Parenting and Family Adjustment Scale and Alabama Parenting Questionnaire highlighted the frequent use of positive strategies, such as positive encouragement.

### Methodological Quality of Included Studies

Overall, the methodological quality of the 12 included studies was rated as being high (n = 8) or moderate quality (n = 4; see Table 4). However, 4 quantitative studies did not state their response rate, so the risk of non-response bias and representativeness of their sample was unclear. The quality of qualitative studies was slightly stronger, with all three scoring 100% and rated to be of high quality. However, qualitative studies often align better with some of the MMAT criteria and therefore score more highly when compared to quantitative studies, so it should not be concluded that the quantitative studies were weaker methodologically.

**Table 4:** Methodological quality assessment of included 12 studies

Authors (year)	Screening questions		Quantitative descriptive domains					Total score (quality appraisal)
	Are there clear research questions?	Do the collected data allow to address the research questions?	Is the sampling strategy relevant to address the research question?	Is the sample representative of the target population?	Are the measurements appropriate?	Is the risk of nonresponse bias low?	Is the statistical analysis appropriate to answer the research question?	
Campbell et al. (2018)	Yes	Yes	Yes	Yes	No	Can’t tell	Yes	Moderate (60%)
Campbell et al. (2012)	Yes	Yes	Yes	Yes	No	Yes	Yes	High (80%)
Mowbray et al. (2002)	Yes	Yes	Yes	Can’t tell	Yes	Yes	Yes	High (80%)
Zemencuk et al. (1995)	Yes	Yes	Can’t tell	Can’t tell	Yes	Yes	Yes	Moderate (60%)
Wolfenden et al. (2022)	Yes	Yes	Yes	Yes	Yes	Can’t tell	Yes	High (80%)

Authors (year)	Screening questions		Quantitative non-randomised					
	Are there clear research questions?	Do the collected data allow to address the research questions?	Are the participant’s representative of the target population?	Are measurements appropriate regarding both the outcome and intervention (or exposure)?	Are there complete outcome data?	Are the confounders accounted for in the design and analysis?	During the study period, is the intervention administered as intended?	
Sabih et al. (2023)	Yes	Yes	Yes	Yes	Yes	Yes	Yes	High (100%)
Rabha et al. (2021)	Yes	Yes	Can’t tell	Yes	Yes	Yes	Yes	High (80%)
Gregg et al. (2021)	Yes	Yes	Can’t tell	Yes	Yes	No	Yes	Moderate (60%)
Shenoy et al. (2019)	Yes	Yes	Can’t tell	Yes	Yes	Yes	Yes	High (80%)

Authors (year)	Screening questions		Qualitative domains					Quality appraisal
	Are there clear research questions?	Do the collected data allow to address the research questions?	Is the qualitative approach appropriate to answer the research question?	Are the qualitative data collection methods adequate to address the research question?	Are the findings adequately derived from the data?	Is the interpretation of results sufficiently substantiated by data?	Is there coherence between qualitative data sources, collection, analysis and interpretation?	
Boström and Strand (2021)	Yes	Yes	Yes	Yes	Yes	Yes	Yes	High (100%)
Strand et al. (2020)	Yes	Yes	Yes	Yes	Yes	Yes	Yes	High (100%)
Graham and King (2005)	Yes	Yes	Yes	Yes	Yes	Yes	Yes	High (100%)

## Discussion

This systematic review is the first to synthesise both quantitative and qualitative research exploring and investigating the parenting practices of parents with psychosis, with findings categorised by research question.

A comprehensive systematic search was carried out, and data were synthesised from 12 quantitative and qualitative studies, based on 1115 parents with psychosis and their parenting practices across six countries, over 28 years. The majority (75%) of included studies utilised quantitative methods, and although this limits a deeper understanding of how parents with psychosis experience parenting and describe their parenting, the quantitative studies used six different data collection tools to assess parenting practices, allowing for a breadth of different aspects of parenting practices to be investigated.

Five of these measures have been found to be valid and reliable tools to assess parenting, but the studies by Campbell et al., (2012, 2018) utilised their own survey and interview, and reliability and validity are therefore unclear. Whilst most subscales in this review could be grouped into a broad parenting style, the variation in measures could account for some of the differences found within the results. Likewise, a range of other factors that were not reliably documented in the included studies could have affected fundings. For example, duration and severity of psychosis, and whether the parent developed psychosis before or after having children was rarely reported. For the majority, study designs were cross-sectional and there is evidence that parenting may change over time in relation to symptoms (Kahng et al., 2008), particularly in psychotic disorders in which relapse is common.

Nonetheless, some reasonably robust conclusions can still be made. The evidence suggests that parents with psychosis often employed positive authoritative parenting strategies, with large-scale studies indicating that the majority of parents report no dysfunction in their parenting 76.6% (Campbell et al., 2012). Although only seven studies measured or explored positive aspects of parenting, those that did, highlighted that parents with psychosis often utilised this parenting style. When the utilisation of positive parenting strategies was compared to parents without SMI, results were inconsistent. Some studies found that parents with psychosis were significantly less likely to use positive parenting strategies than parents without SMI (Rabha et al., 2021; Sabih et al., 2023), whereas Gregg et al. (2021) found no difference between the groups.

Permissive and inconsistent parenting was also commonly reported within both quantitative and qualitative studies, indicating challenges in setting limits and implementing rules and boundaries. All four studies that utilised a control group found that parents with psychosis were significantly more likely to use permissive and lax parenting practices when compared to parents without SMI (Gregg et al., 2021; Rabha et al., 2021; Sabih et al., 2023; Shenoy et al., 2019) aligning with research showing that parents with other mental illnesses such as bipolar disorder are also more permissive in their parenting than controls (e.g. Arman et al., 2018). In agreement with Harries et al.’s (2023b) and Oyserman et al.’s (2000) reviews of parents with SMI, the included studies highlighted the impact of symptoms (hallucinations and delusions), medication side-effects and fatigue on parents’ ability to consistently implement rules and boundaries increasing the likelihood of permissive or authoritarian parenting approaches.

While positive and permissive parenting strategies exhibited more consistent findings across studies, the results for authoritarian parenting were varied, with some studies reporting parents scoring above the clinical range and others below. According to Rabha et al. (2021), parents with psychosis scored the lowest on authoritarian methods, such as corporal punishment, indicating that parents were more likely to use positive authoritative or permissive parenting strategies. Mixed findings were also evident when comparing the use of authoritarian practices among parents with psychosis and without SMI. Additionally, qualitative studies did not explicitly explore or comment on the use of authoritarian practices, therefore, it is difficult to fully understand the extent to which parents with psychosis engage in authoritarian parenting.

Research suggests that psychosis might impact parenting in several ways. Parenting can be stressful for all parents, but according to Zubin and Spring’s (1977) stress vulnerability model, individuals with inherent vulnerabilities, like parents with psychosis, are more prone to stressors, making carrying out the parenting role more difficult. Furthermore, factors such as parenting self-efficacy, parental satisfaction, competence and attitude, which have all been found to be lower in parents with psychosis, have been associated with more permissive and authoritarian parenting more generally (Gelkopf & Jabotaro, 2013; Oyserman et al., 2002).

Psychosis symptoms such as hallucinations, delusions, and disorganised thinking, can affect an individual's ability to respond consistently (National Institute for Health and Care Excellence, 2014). Shenoy et al. (2019) also found a significant association between negative symptoms and parental laxness. These findings suggest a need for targeted interventions that address the specific challenges faced by parents with psychosis, supporting them in maintaining positive and effective parenting strategies despite their mental health difficulties.

### Strengths, Limitations and Recommendations

The parent samples were diverse in terms of ages of children and whether they lived within a dual or single-parent household, allowing for a greater and broader understanding of parental practices for parents with psychosis, representing a strength of the review. No differences were observed between studies in relation to child age, family composition or culture, suggesting that the findings may be applicable across a range of families. However, five studies (42%) only recruited mothers and fewer than half (42%) of the studies reported on ethnicity, questioning the generalisability and transferability of findings to fathers and across ethnic groups. Due to the lack of parenting research conducted with fathers and the higher prevalence of poorer mental health outcomes for ethnic minority groups (Maura & Weisman de Mamani, 2017), it is recommended that future research should include fathers as well as mothers and report on and explore ethnicity.

Only a small number of studies (33%) utilised a control group, limiting our understanding of the similarities and differences in the parenting practices of parents with psychosis compared to parents without SMI. However, studies of parents with bipolar disorder provide evidence of more permissive and authoritarian parenting when compared to controls (e.g. Arman et al., 2018) indicating the need for more research comparing parents experiencing psychosis to those with other mental illnesses and to those without to better understand and identify the support needs of parents with psychosis. Such research should also seek to determine the severity of psychosis symptoms and explore the impact of psychosis symptoms on parenting over time.

Given the small number of qualitative studies and the aims of these studies not solely focusing upon parenting practices, it meant that everyday parenting was not explored in detail, instead focusing on a specific parenting task (e.g. bedtime), and the experience and impact of parental mental health on their parenting. Future research exploring or observing the specific aspects of the parent’s day-to-day parental practices such as routines and implementation of rules/boundaries, with both parents with and without psychosis would again be helpful to gain a deeper understanding of how parents with psychosis parent and identify any similarities or differences to parents without SMI. Future research should also seek to minimise the effects of social desirability bias likely to be evident in explorations of parenting, given reports of stigma and self-criticism in parents with mental illness (Yates & Gatsou, 2021).

The current review was limited to peer-reviewed papers published in English, due to language constraints and lack of funding for translation. However, as only two studies were excluded based on language (Dubreucq et al., 2022; Paraskevoulakou & Schauder, 2020), their exclusion was not considered to affect the overall analysis. The integrative nature of this review allowed for an in-depth understanding of what research has been carried out to investigate and explore the parenting practices of parents with psychosis. Steps were taken to ensure methodological rigor, to imbed transparency and to reduce bias at all stages of study screening, selection and quality appraisal. All included studies were also all rated as being of high or moderate methodological quality, enhancing credibility and trustworthiness of the review findings.

### Clinical Implications

The current review highlights that most parents with psychosis are parenting well and employing positive authoritative parenting practices. However, some differences in parenting practices in parents with psychosis were noted, specifically a tendency toward permissive parenting, potentially due to the negative impact of the additional challenges parents with psychosis reported including fatigue, medication side-effects and mental health symptoms (Graham & King, 2005; Strand et al., 2020). Baumrind’s (1966) theory of parenting styles suggests that such difficulties create a less predictable environment and increase the likelihood of parents adopting more permissive or authoritarian styles. Parenting interventions focusing on more adaptive parenting skills may be beneficial to help increase parental confidence in implementing more positive and authoritative strategies when parents are feeling overwhelmed. Furthermore, acknowledging and encouraging the use of positive parenting strategies that parents are already employing through a strength-based framework may positively impact both parent and child outcomes. One study included within this review implemented a parenting programme for parents with psychosis and measured practices at baseline and post-intervention (Wolfenden et al., 2022). It identified a significant decrease in dysfunctional discipline practices after the intervention (decreased permissiveness and over-reactivity on the PS). Wolfenden et al. (2022) reported that all parents fell below the clinical range of the PS following the Triple P positive parenting programme, highlighting the benefits of structured support for parents with psychosis.

Considering the significant role of the parent on child outcomes (Davidsen et al., 2022; Dean et al., 2010; Somers, 2007), it is important that healthcare professionals working with parents with psychosis have an awareness of the difficulties they may experience, and the support options available. This understanding will hope to improve better signposting to supportive services. Aligned with previous recommendations advocating for increased support for these families (Bee et al., 2014; Diggins, 2011; Radley et al., 2022b), this review highlights the need for further development of family-focused approaches, to promote conversations in services regarding the challenges and successes of parenting. Although recommendations are in place by clinical organisations, to ensure adult mental health services are recognising the parenting and familial role of individuals (Care Act, 2014; Diggins, 2011; Royal College of Psychiatrists, 2011), research by Dunn et al. (2022) highlighted that less than half of adult mental health practitioners engage with the parenting experience and the potential impact of parents’ mental health on children. Given the substantial impact psychosis can have on the whole family (Abel et al., 2019; Davidsen et al., 2022; Pierce et al., 2020), interventions adopting a family-focused approach and mental health practitioners’ discussing the parental role can help to mitigate the risks associated with parental mental health difficulties.

### Conclusion

This was the first mixed methods review to comprehensively synthesise research exploring and investigating the parenting practices of parents with psychosis. Parents with psychosis often employed positive parenting strategies but were also more likely to use permissive practices when compared to parents without SMI. This review provides valuable insights into the everyday parenting practices of parents with psychosis, enhancing our understanding of their family life and highlighting the need for further support and future research. In light of the findings, it is important to increase healthcare professionals’ awareness of the challenges faced by parents with psychosis, alongside improving support for parents and families through family-focused approaches and the implementation of evidence-based parenting interventions. These recommendations may serve to improve outcomes for both parents with psychosis and their children and families.

## Data Availability

The authors confirm that all data generated or analysed during this study are included in this published article.

## Footnotes

1 1 It is possible that two studies by the same research group (Campbell et al., 2012, 2018) contain some of the same parents.
